# Developments on drug discovery and on new therapeutics: highly diluted tinctures act as biological response modifiers

**DOI:** 10.1186/1472-6882-11-101

**Published:** 2011-10-26

**Authors:** Carolina C de Oliveira, Ana Paula R Abud, Simone M de Oliveira, Fernando de SF Guimarães, Lucas F de Andrade, Raffaello P Di Bernardi, Ediely L de O Coletto, Diogo Kuczera, Eneida J Da Lozzo, Jenifer P Gonçalves, Edvaldo da S Trindade, Dorly de F Buchi

**Affiliations:** 1 Laboratório de Estudos de Células Inflamatórias e Neoplásicas, Departamento de Biologia Celular, SCB, Centro Politecnico, Universidade Federal do Paraná - Curitiba, CEP 81531-980, PR - Brazil

**Keywords:** drug discovery, highly diluted tinctures, immune cells, bone marrow cells, lymph node cells, macrophages

## Abstract

**Background:**

In the search for new therapies novel drugs and medications are being discovered, developed and tested in laboratories. Highly diluted substances are intended to enhance immune system responses resulting in reduced frequency of various diseases, and often present no risk of serious side-effects due to its low toxicity. Over the past years our research group has been investigating the action of highly diluted substances and tinctures on cells from the immune system.

**Methods:**

We have developed and tested several highly diluted tinctures and here we describe the biological activity of M1, M2, and M8 both *in vitro *in immune cells from mice and human, and *in vivo *in mice. Cytotoxicity, cytokines released and NF-κB activation were determined after *in vitro *treatment. Cell viability, oxidative response, lipid peroxidation, bone marrow and lymph node cells immunophenotyping were accessed after mice *in vivo *treatment.

**Results:**

None of the highly diluted tinctures tested were cytotoxic to macrophages or K562. Lipopolysaccharide (LPS)-stimulated macrophages treated with all highly diluted tinctures decreased tumour necrosis factor alpha (TNF-α) release and M1, and M8 decreased IFN-*γ *production. M1 has decreased NF-κB activity on TNF-α stimulated reporter cell line. *In vivo *treatment lead to a decrease in reactive oxygen species (ROS), nitric oxide (NO) production was increased by M1, and M8, and lipid peroxidation was induced by M1, and M2. All compounds enhanced the innate immunity, but M1 also augmented acquired immunity and M2 diminished B lymphocytes, responsible to acquired immunity.

**Conclusions:**

Based on the results presented here, these highly diluted tinctures were shown to modulate immune responses. Even though further investigation is needed there is an indication that these highly diluted tinctures could be used as therapeutic interventions in disorders where the immune system is compromised.

## Background

Highly diluted substances and tinctures are usually intended to enhance the immune system resulting in reduced frequency of various diseases. The immune system is a complex network that integrates lymphoid organs, cells, humoral factors, and cytokines. The essential function of the immune system is host defense, but when it does not work properly the disease emerges. For instance, underactivity may result in severe infections and tumors of immunodeficiency, overactivity in allergic and autoimmune disease [1]. When the body successfully fights any potential harmfully agent by itself it results in an enhancement of the immune system. Conventional treatments often are directed to terminate the symptoms rather than fighting the disease cause, and frequently present side effects. Adverse drug reactions inflict serious injury to million people around the world. It is known that all substances can produce harmful effects associated with its toxic properties, and quoting Paracelsus (1493-1541) "all substances are poisons; there is none which is not a poison. The right dose differentiates a poison". The search for effective treatments with low toxicity to patient is needed. Changes in the dose could be effective in order to diminish the harmful side effects. All drugs present a dose response curve, or a range of doses that result in a graded effect between the extremes of no effect and 100% response which is the toxic effect. If the dose is low enough even a highly toxic substance will cease to cause the harmful effect on biological systems. Thus in the case of using highly diluted compounds or tinctures you might avoid the harmful effects as it often present no risk of serious side-effects because of their low toxicity, and still obtain an effective response such as modulation of the immune system.

Over the past years our research group has been testing the action of highly diluted substances and tinctures on cells from the immune system [2-4]. The development of new combinations was based on laboratorial tests and assays were performed independently and in a blind manner. These were crucial to determine which substances to use on each combination of highly diluted tinctures. For instance, one substance when used alone presents modification in biological responses, i.e. it increases cytokine production by cells, but when combined to another substance the effects on biological responses are annulled (data not published yet). Biological assays are intended to understand better what happens within a cell when a molecule interacts with its target. In order to determine efficacy and safety of new drugs there is a clear move within the pharmaceutical industry towards increased emphasis on cell-based assays. Well-structured methodological studies are useful to understand how highly diluted tinctures work. Thus we have developed and tested several new highly diluted tinctures, of which three of them are described here. As products were coded by an external observer, they were initially coded as M followed by a number. In the present study we describe the biological activity of M1, M2 and M8 both *in vitro *in immune cells from mice and human, and *in vivo *in mice.

## Methods

### 2.1. Combination of highly diluted tinctures

Mother tinctures were produced by Laboratório Schraibmann Ltda (Carapicuíba - Brazil) which is authorized by the Brazilian Health Ministry. They certify high quality, physicochemical composition, and endotoxin-free conditions of its products. Dilution procedures have followed standard methodology described at the Brazilian Homeopathic Pharmacopoeia. In brief, starting from a whole extract (MT) or initial decimal dilutions (dH) of several tinctures, the highly diluted tinctures were obtained by serial decimal dilutions in distilled water (1 part of tincture to 9 parts of water - v/v). In between each dilution, the solution was vigorously manual shaken (by hand) with 100 strong vertical strokes against a soft pad. The resulting aqueous solution is colorless and odorless, and contains 0.1% alcohol. These were stored in the dark at room temperature. Three complex products were used, namely M1, M2 and M8. Its composition is described in table 1 together with the original concentration of the matrices.

**Table 1:** Composition of highly diluted tinctures with initial and final dilution of each element.

Components	Matrix	Final concentration			Final concentration (v/v %)
		M1*	M2*	M8*	

***Aconitum napellus***	MT	20 dH	20 dH	20 dH	0.1 × 10^-19^

***Arsenicum album***	6 dH	18 dH	18 dH	18 dH	0.1 × 10^-17^

***Asa foetida***	MT	20 dH	20 dH	20 dH	0.1 × 10^-19^

***Calcarea carbonica***	8 dH	16 dH	16 dH	16 dH	0.1 × 10^-15^

***Chelidonium majus***	MT	20 dH	20 dH	-	0.1 × 10^-19^

***Cinnamon***	MT	20 dH	20 dH	-	0.1 × 10^-19^

***Conium maculatum***	5 dH	17 dH	17 dH	17 dH	0.1 × 10^-16^

***Echinacea purpurea***	MT	20 dH	-	-	0.1 × 10^-19^

***Gelsemium sempervirens***	MT	20 dH	-	-	0.1 × 10^-19^

***Ipecacuanha***	5 dH	13 dH	13 dH	13 dH	0.1 × 10^-12^

***Phosphorus***	12 dH	20 dH	20 dH	20 dH	0.1 × 10^-19^

***Rhus toxicodendron***	6 dH	17 dH	17 dH	17 dH	0.1 × 10^-16^

***Silicea***	12 dH	20 dH	20 dH	20 dH	0.1 × 10^-19^

***Sulphur***	12 dH	24 dH	24 dH	24 dH	0.1 × 10^-23^

***Thuja occidentalis***	6 dH	19 dH	19 dH	19 dH	0.1 × 10^-18^

*M1, M2, and M8 were prepared from 15, 13, and 11 components, respectively, in different dilutions. In-between dilutions, the solutions are vigorously shaken by hand against a soft pad.

### 2.2. *In vitro *determination of M1, M2 and M8 biological activity on human cell lines

HT29 (ATCC: HTB-38) derived from human colon-rectal cancer, K562 (ATCC: CCL- 243) immortalized myelogenous leukemia cells from human, monocytes and monocytes derived macrophages from human donors were used in *in vitro *experiments, that were performed at 37°C in a humidified 5% CO2 atmosphere for 48 hours. All treatments were administered to log-phase of growing cells. All solutions used to treat cells were vigorously shaken before adding them to cell culture. An initial dose of 20% (v/v) of highly diluted tinctures was added to the cells and, after 24 hours, a reinforcement dose of 1% was added according to a previous standard treatment protocol [2]. All experiments were performed at least three times in a blind manner. Data representative of those experiments (n = 9 for each group) were submitted to ANOVA and Tukey test to determine statistical difference (p < 0.05).

#### 2.2.1. Cytotoxicity assays

Apoptosis and necrosis were estimated after treatment. Immortalized myelogenous leukemia cells from human K562 were treated with M1, M2 and M8 and percentage of cell death was determined by flow cytometry. Annexin V-FITC Apoptosis detection kit (BIOPharmigen) and propidium iodide (PI) were used to evaluate cell viability and DNA content in cell cycle according to manufacture instructions.

#### 2.2.2. Cytokines detection

Human white blood cells were isolated from whole-blood leukoreduction filters obtained after platelet apheresis. They were donated by healthy volunteers at the apheresis center from Hospital de Clínicas - UFPR. These cells were assayed *in vitro *in order to detect cytokine production after treatment. All related practice was approved by the Ethical Committee on Human Research from UFPR.

The content of leukoreduction chamber was eluted with PBS, and peripheral blood mononuclear cells were purified using standard Ficoll-Paque gradient centrifugation. After monocytes purification, cells were resuspended in complete RPMI 1640 medium supplemented with 2.5 ng/ml rhGM-CSF (PeproTech, London, England), and maintained at 37°C, 5% CO_2 _for 9 days to differentiation into macrophages. Cells were dislodged with trypsin and then plated (5 × 10^5 ^cells/well in 96-well tissue culture plates) to determine cytokines release. Macrophages ability to produce cytokines when challenged with each highly diluted tinctures, in the presence or not of 5 μg/mL LPS (lipopolysaccharide) was determined after 48 hours on supernatant of cultures using human Th1/Th2 cytokine CBA kit (BD - Pharmingen) according to manufacture instructions. Fluorescence was detected by flow cytometry and cytokine concentration was obtained comparing data with standards cytokines curve at the CBA program (BD).

#### 2.2.3. HT29-pNFκB-hrGFP reporter cells

Stable transfection with the pNF-κB-hrGFP plasmid (PathDetect signal transduction pathway cis-reporting systems kit - Stratagene) was performed on HT29 (ATCC: HTB-38) cells. Briefly subconfluent HT29 cells were transfected with pNF-κB-hrGFP plasmid using Lipofectamine 2000 (Invitrogen) and selected with hygromycin. After two weeks, cells were stimulated for 24 h with a pro-inflammatory cocktail (25 ng/mL TNFα-tumor necrosis factor alpha, and 1.25 ng/mL IL-1b - interleukin 1 beta). GFP (green fluorescent protein) positive cells were sorted with a MoFlo cell sorter (Dako, Carpinteria, CA).

The sorted cells were maintained in RPMI (GIBCO) containing 10% FBS, 1 U/mL penicillin, 1 μg/mL streptomycin at 37°C in a humidified 5% CO2 atmosphere. For the NF-κB (nuclear factor kappa B) activation assay, exponentially growing HT29 p NF-κB-hrGFP cells were cultured for 48 hours in absence or presence of 3 ng/mL TNFα with or without M1, M2, or M8 products. Percentage of positive GFP cells and the viability (using propidium iodide- PI) were determined using CyAn™ ADP Flow Cytometer (Dako, Carpinteria, CA) and Summit v4.3 software [4].

### 2.3. *In vivo *treatment

#### 2.3.1. Animals

Six to eight weeks old male albino Swiss mice, weighing 30-35 g, were grown and maintained at Central Animal House from Universidade Federal do Paraná (UFPR). They were housed at controlled temperature (22 ± 3°C), 12-hours light/dark cycle, and received a standard laboratory diet. All recommendations from Brazilian National Law n° 6.638.058, Nov. 1979, for scientific management of animals were followed. Institutional Animal Care Committee approved all related practices.

Mice were weight and randomly assembled into four groups containing 10 animals in each. M1, M2 and M8 lacking one decimal dilution along with distilled water (control) were kept in distinct 20 mL glasses that were coded by an external observer. At the moment of treatment, we have added 20 mL into a bottle containing 180 mL of drinking water. All bottles were vigorously shaken and offered to mice just before the beginning of the dark cycle. Each animal has received approximately 10 ml/day for 7 days. Bottles were replaced every day with new one containing freshly diluted solutions.

#### 2.3.2. Biological activity analysis of M1, M2 and M8 on mice cells

At the end of the studies animals were killed by cervical dislocation (n = 9 for each tested group). Macrophages, lymph nodes, and bone marrow cells were removed as described elsewhere [2-4]. In brief, macrophages were harvested from the mice peritoneal cavities with cold phosphate buffer saline (PBS), pH 7.4, and cells were counted using a Neubauer chamber. Lymph nodes were removed from the mesentery, tissue was dissociated using sterile Medicons (BD), and the cell suspension was filtered with a 100-μm mesh filter (Falcon, BD). Cells were pelleted by centrifugation, resuspended in PBS, incubated in a culture flask with PBS at 37°C in a humidified atmosphere containing 5% CO2. After incubating for 40 min, non-adherent cells were transferred to sterile tubes, washed three times with PBS and counted using an automated cell counter (CELM). Femurs were dissected and cleaned. Epiphyses were removed and the marrow was flushed with Dulbecco's Modified Eagles Medium (DMEM) containing 10% fetal bovine serum (FBS) with 1 U/ml penicillin, 1 mg/ml streptomycin, and 2.5 mg/ml amphotericin. All cell types were counted and cell density adjusted depending on the experiment. Cells were then plated and cultured for each experiment.

##### 2.3.2.1. Cytotoxicity assays

The neutral red assay is based on the initial protocol described by Borenfreund and Puerner in 1984 [5] and determines the accumulation of the neutral red dye in the lysosomes of viable, uninjured cells. Following exposure to highly diluted tinctures macrophages were incubated for 2 hours with neutral red dye (10 ug/ml). Cells were then washed, and the ethanolic dissolution medium was added followed by gentle shaking for 10 min so that complete dissolution was achieved. Absorbance at 540 nm was recorded using the microplate reader (ELx 800, Meridian Diagnostic, Inc).

##### 2.3.2.2. Reactive oxygen and nitrogen species

Macrophages were subjected to analyses of reactive oxygen species (ROS) and nitrogen species production. Anion superoxide O_2_^-^, hydrogen peroxide (H_2_O_2_), and nitric oxide (NO) were determined on culture supernatant as described elsewhere [6-8]. Briefly, after *in vivo *treatment, mice were killed and macrophages were removed and cultivated on 96 well plates (5 × 10^5 ^cells/well) at 37°C in a humidified atmosphere containing 5% CO_2_. After 15 min, non-adherent cells were removed by washing. The remaining adherent cells, mainly macrophages, were incubated with the specific reaction solution for each assay for different periods. Resulting change in absorbance was determined in a microplate reader (ELx 800, Meridian Diagnostic, Inc). Proper standard concentration curves and positive controls were also done according to previous published methodology [6-8].

##### 2.3.2.3. Lipid peroxidation and immunophenotyping of bone marrow cells

Bone marrow cells were collected from femurs and immunophenotyped using a mouse lineage panel as described elsewhere [3]. In brief, one million cells per group were fixed with 1% paraformaldehyde, washed, and incubated with 0.5 μg/ml biotinylated antibody listed below, in PBS for 40 minutes. They were then washed with PBS and incubated with 0.5 μg/ml phycoerythrin (PE) labeled secondary antibody in PBS for 30 minutes. Fluorescence was analyzed according to standard procedures using a FACSCalibur flow cytometer.

Lipid peroxidation was determined according to Nourooz-Zadeh and colleagues in 1994 [9]. Briefly, ten million bone marrow cells were homogenized in methanol, centrifuged and incubated for 30 minutes with 10 mM triphenylphosphine (TPP) solution. After cells were incubated with FOX2 (100 μM xylenol orange, 400 mM butylated hydroxytoluene, 25 mM sulfuric acid and ammonium ferrous sulphate in 90% methanol was added and incubated at RT for 30 min. Absorbance was determined on a spectrophotometer at 560 nm. Data was obtained compared to standard curve.

##### 2.3.2.4. Immunophenotyping of lymph nodes cells

Lymph node cells were isolated as described above. Cells (10^6 ^cells/sample) were incubated with anti-CD3/FITC and anti-CD4/PE, anti-CD8/PE, anti-CD19/PE or Pan-NK/PE (BD Pharmingen) antibodies in PBS/1%FBS for 30 min, and washed three times with PBS. Results were obtained by flow cytometry using CellQuest software (BD) [4].

### 2.4. Statistical analysis

All *in vitro *and *in vivo *experiments were performed at least three times in triplicate, and data analysis was performed in a double-blinded manner. All data generated were submitted to analysis of variance (ANOVA) and Tukey test. ANOVA is a statistical test that search for a difference across groups as a whole. If there is a statistically significant difference across means then a multiple comparison method should be used to try to find specific differences between pairs of groups. In this case we have used Tukey test to determine the statistical significance of the intergroup comparisons (p < 0.05).

## 3. Results

### 3.1. Determination of *in vitro *M1, M2 and M8 biological activity on cytotoxicity, cell cycle, cytokine production, and NF-κB activation

**Figure 1:**
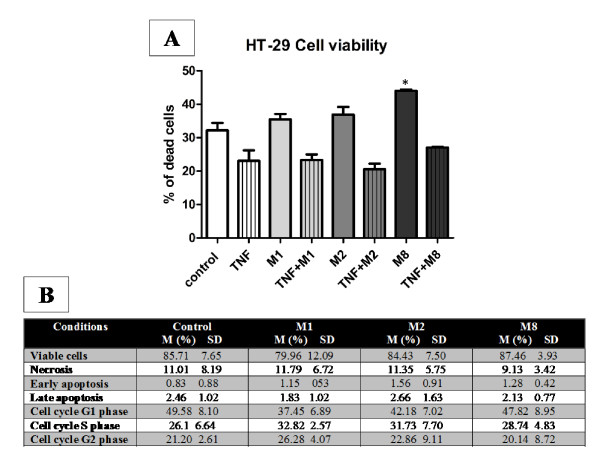
**Effects of treatments on HT-29 and K562 cells viability and cell cycle**. Apoptosis and necrosis were estimated after treatment. HT29-pNF-κB-hrGFP reporter cells HT29 (Fig A) and immortalized myelogenous leukemia cells from human K562 (Fig B) and were treated with M1, M2 and M8 and percentage of cell death was determined by flow cytometry. Annexin V-FITC Apoptosis detection kit (BIOPharmigen) and propidium iodide (PI) were used to evaluate cell viability and DNA content in cell cycle. ANOVA and Tukey test were use to determine statistical differences. Neither highly diluted compound were cytotoxic nor have effects on cell cycle. *p < 0.05

Annexin V is often used to study the translocation of phosphatidylserine (PS) from the inner leaflet of the plasma membrane to the outer leaflet in apoptotic cells. It is a simple and effective method to detect apoptosis at even very early stages [10]. Propidium iodide (PI) intercalates into double-stranded nucleic acids. It is excluded by viable cells but can penetrate cell membranes of dying or dead cells. Thus double-labeling with annexin V and PI could be used to differentiate between different types of cell death. The neutral red assay is used to measure cell viability, as living cells take up the neutral red, which is concentrated within the lysosomes of cells [11]. We have observed a tendency of cell death after M8 treatment on HT-29 reporter cell line after statistical analyses at p < 0.05 level (Figure 1A). None of the highly diluted tinctures tested were cytotoxic to macrophages (data not shown), and K562 (Figure 1B). Results also demonstrated that M1, M2, and M8 have no effects on cell cycle (Figure 1B). Although M8 induced cell death on HT-29 cells all other cells tested were not affected by M8, including B16F10 cells [4].

**Figure 2:**
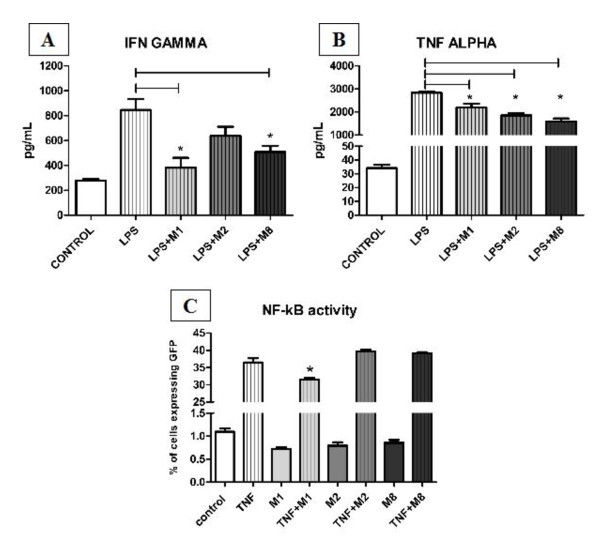
**Cytokine production and NF-κB activation assay after M1, M2, and M8 treatment**. Fig A - Human monocytes were isolated and differentiated into macrophages. Macrophages ability to produce interferon-γ (IFNγ) and tumor necrosis factor (TNF-α) when challenged with each highly diluted tincture, in the presence or not of 5 μg/mL LPS was determined after 48 hours on supernatant of cultures using CBA. Fluorescence was detected by flow cytometry, and ANOVA and Tukey test were use to determine statistical differences.*LPS-induced macrophages decreased IFN production after M1 and M8 treament and TNF-α release was decreased by all medications. Fig B - Exponentially growing HT29 p NF-κB-hrGFP cells were cultured for 48 hours in absence or presence of 3 ng/mL TNFα with or without M1, M2, or M8 highly diluted tinctures. Percentage of positive GFP cells was determined by flow cytometery. M1 treatment after TNF stimulation has decreased NF-κB activity, and different concentrations of M1 had the same effects. *p < 0.05

Human monocytes were differentiated into macrophages and treated with M1, M2, and M8 in the presence or absence of LPS. There was no change in cytokines release with treatment alone. LPS-stimulated macrophages treated with M1, and M8 presented a decrease in interferon-*γ *(IFN-*γ*) production, and M1, M2, and M8 a decrease in tumor necrosis factor-α (TNF-α) release (Figure 2A and 2B).

HT29-pNF-κB-hrGFP reporter cells were used to determine NF-κB after treatment. We have observed that only M1 has decreased NF-κB activity on TNF-α stimulated HT29-pNF-κB-hrGFP cells (Figure 2C). Thus we have tested *in vitro *different M1 concentrations (10, 20, ad 30%) and they all presented the same effect on NF-κB activity (data not shown).

### 3.2 Biological activity of *in vivo *administration of M1, M2 and M8 to mice

**Figure 3:**
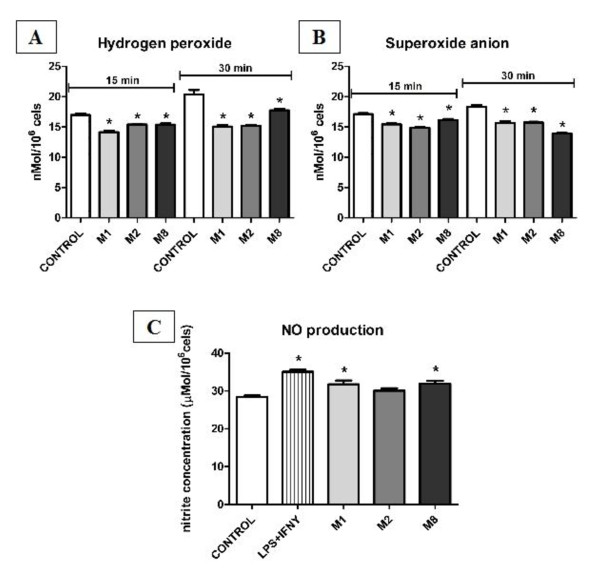
**ROS and NO production by macrophages after treatment**. Macrophages were subjected to analyses of reactive oxygen species (ROS) and nitrogen species production. Macrophages were incubated with the specific reaction solution for each assay for different periods. Resulting change in absorbance was determined in a microplate reader. Proper standard concentration curves and positive controls were also done. ANOVA and Tukey test were use to determine statistical differences. We have found that the levels of superoxide anion (O_2_^-^) and hydrogen peroxide (H_2_O_2_) release after all treatments have decreased. NO was found to be increased after M1 and M8 treatments. *p < 0.05

**Figure 4:**
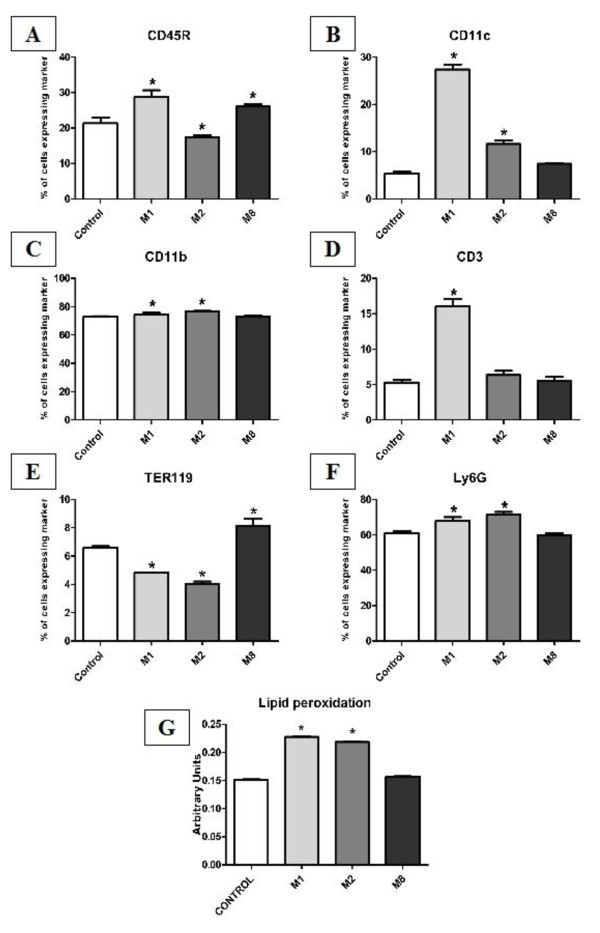
**Immunophenotyping and lipid peroxidation of mice bone marrow cells**. Mice were treated for 7 days, femurs were removed and bone marrow cells were collected. Cells were immunophenotyped using a mouse lineage panel (Fig A-F). Fluorescence was analyzed using flow cytometry and data were analyzed with a Cell Quest program (BD). ANOVA and Tukey test were use to determine statistical differences. Treatment with all highly diluted tinctures changed the expression profile of bone marrow cell markers. Lipid peroxidation was determined by FOX2 absorbance on a spectrophotometer at 560 nm (Fig G). Data was obtained compared to standard curve. We have observed lipid peroxidation on bone marrow cells induced by M1 and M2. *p < 0.05

Macrophages, bone marrow and lymph node cells from mice were analyzed after 7 days treatment. All treatments have decreased the levels of superoxide anion O_2_^- ^and hydrogen peroxide (H_2_O_2_) release by macrophages (Figure 3A and 3B). NO was found to be increased after M1 and M8 treatments (Figure 3C). We have observed lipid peroxidation on bone marrow cells induced by M1 and M2 (Figure 4G).

**Figure 5:**
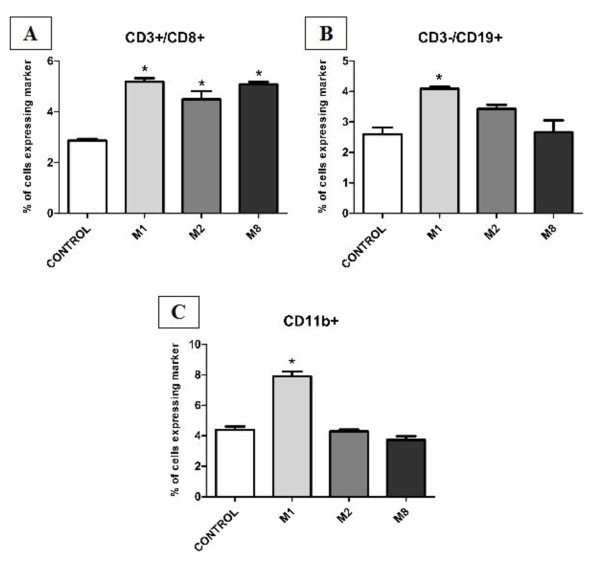
**Immunophenotyping of mice lymph node cells**. Lymph node cells were isolated and immunophenotyped. Results were obtained by flow cytometry using CellQuest software (BD). ANOVA and Tukey test were use to determine statistical differences. Treatment with all highly diluted tinctures has changed the expression profile of lymph node cell markers. *p < 0.0

A mouse lineage panel was used to immunophenotype bone marrow cells (CD11b (Mac-1)-monocytes/macrophages, Ly-6G-granulocytes, CD45R-B lymphocytes, CD11c-dendritic cells, CD3-T lymphocytes, and TER-119-erythrocytes), as well to lymph node cells (CD11b-Monocytes/macrophages, CD3-T lymphocytes, CD4-T helper lymphocytes, CD8-T cytotoxic lymphocytes, CD19-B lymphocytes, PanNK (DX5)-NK lymphocytes). Immunophenotyping of bone marrow (Figure 4A-F) and lymph node cells (Figure 5A-C) showed that each composition of highly diluted tinctures presented a different pattern of host immune response. M1 has increased monocytes/macrophages and B lymphocytes in bone marrow and lymph nodes. CD3 lymphocytes and granulocytes were increased and erythrocytes were decreased in bone marrow. M2 has increased monocytes/macrophages and granulocytes and decreased B lymphocytes as well as erythrocytes in bone marrow. And M8 has increased B lymphocytes in bone marrow. Lymph nodes T cytotoxic lymphocytes were increased in lymph nodes by all treatments. Table 2 summarizes the biological activity analysis of M1, M2 and M8 on mice cells.

**Table 2:** Summary of the biological activity analysis of M1, M2 and M8 on mice cells.

Macrophages				Bone marrow cells		Lymph node cells	General comments
	**O_2_^-^**	**H_2_O_2_**	**NO**	**Lipid peroxidation**	Immunophenotyping*	**Immunophenotyping^+^**	**Immune response type**

**M1**							

**M2**							

**M8**							

*Detection of changes in cell markers on bone marrow cells after treatment. + Detection of specific cell markers on lymph node cells. Grey arrow - decrease in production or detection; black arrow - increase in production or detection; O_2_^- ^- superoxide anion; H_2_O_2 _- hydrogen peroxide; NO - nitric oxide.

## Discussion

Plant extracts are common subjects of basic research. For instance, imunopharmacological potential of *Thuya occidentalis *was widely demonstrated both *in vivo *and *in vitro *as reviewed by Naser and colleagues in 2005 [12]. Highly diluted tinctures or substances are less studied, but there is an increased interest in these type of formulations due to its low cost and biological effectiveness. Bellavite and col. revised in 2006 [13] articles containing basic research on cells of the immune system and inflammation. They have shown that there are few and rather small groups working on laboratory models, although evidences of the biological activity *in vitro *of highly diluted-dynamized solutions is slowly accumulating. Our group has tested some highly diluted tinctures alone and we have found that they present different responses when different potencies are tested (data not published yet). Here we have investigated the effects of three combinations, namely M1, M2, and M8 on mice and human cells. M1, M2, and M8 are prepared from 15, 13, and 11 highly diluted components, respectively, in different dilutions as seen on table 1. We have observed that all these induced different effects on cells, as well as different immune response type, even though they have some similarity on its composition as seen on table 1.

In order to evaluate M1, M2 and M8 safety we have first determined cell viability and cell cycle after *in vitro *treatment. We have used K562, HT-29 cell line and macrophages to determine if treatment could induce cell death and/or changes in cell cycle. We have observed that none of the tested combinations of highly diluted tinctures were cytotoxic or affected cell cycle. Next we sought to determine if there was any effect of these highly diluted tinctures on cytokine release by monocytes-derived macrophages. Treatment alone did not change cytokines production by cells, but when cells were stimulated with LPS and then treated IFN-γ and TNF-α production was decreased. TNF-α is an important inflammatory factor that acts as a master switch in establishing an intricate link between inflammation and cancer. TNF-α secretion can be induced by conserved structural elements common to microbial pathogens as well as by tumour cells. Several studies have focused on the transcriptional regulation of TNF-α looking at transcription factors that bind to the responsive element sites within the TNF-α promoter. NF-κB is a transcription factor that plays crucial roles in inflammation and immunity [14]. Many proinflammatory stimuli can activate NF-κB, mainly through IKK-dependent phosphorylation and degradation of the IκB inhibitory proteins. When NF-κB translocates to the nucleus, it activates the transcription of target genes, including cytokines like TNF-α, chemokines, and antiapoptotic factors [15]. We have used a reporter cell line to find out if M1, M2, and M8 in the presence or absence of TNF-α stimulus, have any effect on NF-κB activity. The reporter cell line HT29-pNF-κB-hrGFP is routinely used to screen natural or synthetic compounds that interfere and/or modulate NF-κB activity. We have observed that only M1 has decreased NF-κB activity but it was not downregulated by M2 and M8. However we have observed TNF-α reduction by these highly diluted tinctures. NF-κB, C/EBPβ, Ets, NF-AT, activating protein 1 (AP-1), cAMP response element-binding protein, signal transducers and activators of transcription (STAT1), and lipopolysaccharide (LPS)-induced TNF-α factor (LITAF) were all implicated on regulating TNF-α transcription [16]. Regulatory elements in transcriptional activation of the TNF-α gene are not completely understood. Further investigation of others transcription factors that regulate TNF-α induction may be conducted in order to investigate the relative contributions of these various regulatory elements in transcriptional decrease of the TNF-α by M2 and M8. Improving the efficacies of anti-inflammatory mediators hold promises for the therapy of cancers and chronic inflammatory disorders. Thus after detecting that M1, M2, and M8 have *in vitro *effects we have established the biological activity of those highly diluted tinctures after mice treatment (*in vivo*).

First we have detected changes on oxidative metabolism of macrophages after M1, M2, and M8 *in vivo *administration to mice. The phagocytic oxidative burst is a primary effector of innate immunity that protects against infection. When triggered by a phagocytic stimulus or certain membrane-active agents, macrophages activated *in vivo *or *in vitro *undergo a respiratory burst that is characterized by the production of high levels of oxygen metabolites. The NADPH-dependent oxidase (Phox), which produces superoxide, assembles in the phagosomal membrane [17]. Activated macrophages also produce nitric oxide (NO), generated from arginine and oxygen by the inducible nitric oxide synthase (iNOS) [18]. Nitric oxide is a cytotoxic product of activated macrophages, along with reactive oxygen species that have been shown to be involved in numerous regulatory functions [19]. Reactive oxygen species have been increasingly implicated as playing a central role in the pathophysiology of clinical infections. More specifically superoxide, hydrogen peroxide, and recently, nitric oxide are thought to contribute to these processes. As these highly diluted tinctures exhibit the ability to modulate the respiratory burst and NO production they could be useful adjutants to host defence and to microbicidal activity [20].

4-Hydroxynonenal (4-HNE) is a product derived from lipid peroxidation that can affect several mechanisms on the cell depending on its concentration. Low levels were shown to induce cell differentiation, even though the exact mechanism of action is still not known [21,22]. In fact, previous results from our lab have shown that highly diluted tinctures act on bone marrow cells stimulating proliferation and differentiation [3]. Thus this might account as one the mechanism of action by how highly diluted tinctures stimulate bone marrow cells proliferation and differentiation, but further experiments are needed.

Bone marrow is a special, spongy, fatty tissue that houses stem cells, located inside a few large bones. These stem cells transform themselves into leukocytes, red blood cells and platelets, essential for immunity and circulation. A mouse lineage panel to bone marrow cells was used (CD11b (Mac-1)-monocytes/macrophages, Ly-6G-granulocytes, CD45R-B lymphocytes, CD11c-dendritic cells, CD3-T lymphocytes, and TER-119-erythrocytes), as well to lymph node cells (CD11b-Monocytes/macrophages, CD3-T lymphocytes, CD4-T helper lymphocytes, CD8-T cytotoxic lymphocytes, CD19-B lymphocytes, PanNK (DX5)-NK lymphocytes). We found that each highly diluted tincture presented a different pattern of host immune response. M1 has increased monocytes/macrophages and B lymphocytes in bone marrow and lymph nodes. CD3 lymphocytes and granulocytes were increased and erythrocytes were decreased in bone marrow. M2 has increased monocytes/macrophages and granulocytes and decreased B lymphocytes as well as erythrocytes in bone marrow. And M8 has increased B lymphocytes in bone marrow. Lymph nodes T cytotoxic lymphocytes were increased in lymph nodes by all treatments. Table 2 summarizes the biological activity analysis of M1, M2 and M8 *in vivo*.

The analysis of highly diluted tinctures after mice treatment showed that M1, M2, and M8 improved the innate (cellular) immunity. In addition, M1 also augmented acquired (humoral) immunity. M2 was the only highly diluted compound that diminished B lymphocytes precursors, which produce antibodies, responsible to humoral immunity. Decrease in antibody production by M2, but without causing immune suppression since it has increased the number of cytotoxic cells, turns out to be interesting to autoimmune diseases where this type of response is extremely important. The results from M8 treatment, as it increases innate immunity and does not alter acquired immunity, suggest a possible use in diseases where antibody production is irrelevant, i.e. viruses where the pathogenic agent is extremely mutable. As for M1 treatment we can conclude that it shows a possible general action on immune system, enhancing both innate and acquired immunity which are important in several diseases including some types of cancers.

## Conclusions

Based on the results presented here, these highly diluted tinctures were shown to modulate immune responses. Even though further investigation is needed there is an indication that these combinations of highly diluted tinctures could be used as therapeutic interventions in disorders where the immune system is compromised. It could be reasonably expected that, in the years to come, there will be a steady growth in the use of highly diluted tinctures as they often present no risk of serious side-effects because of their low toxicity and they are efficacious therapeutic interventions.

## Competing interests

The authors declare that they have no competing interests.

## Authors' contributions

CCO conceived of the study, participated in all experiments from its design to its interpretation, and analyses, as well as draft the manuscript. APRA, SMO, ELOC have carried out the experiments with macrophages and bone marrow both *in vivo *and *in vitro*. FSFG, LFR carried out the *in vivo *experiments with mice, as well as lymphocytes analyses. RPB participated in the design of the study and helped on the statistical analysis. DK, EJL, JPG have designed and carried out the experiments using human cell. EST has provided critical help on experimental design and data analyses. DFB participated in experimental design and analyses, as well as coordination and helped to draft the manuscript. All authors read and approved the final manuscript.

## Pre-publication history

The pre-publication history for this paper can be accessed here:

http://www.biomedcentral.com/1472-6882/11/101/prepub
